# First Record of Ategmic Ovules in Orchidaceae Offers New Insights Into Mycoheterotrophic Plants

**DOI:** 10.3389/fpls.2019.01447

**Published:** 2019-11-29

**Authors:** Mariana Ferreira Alves, Fabio Pinheiro, Marta Pinheiro Niedzwiedzki, Juliana Lischka Sampaio Mayer

**Affiliations:** Departamento de Biologia Vegetal, Instituto de Biologia, Universidade Estadual de Campinas, São Paulo, Brazil

**Keywords:** anatomy, integument, Epidendroideae, saprophytic, Vanilloideae

## Abstract

The number of integuments found in angiosperm ovules is variable. In orchids, most species show bitegmic ovules, except for some mycoheterotrophic species that show ovules with only one integument. Analysis of ovules and the development of the seed coat provide important information regarding functional aspects such as dispersal and seed germination. This study aimed to analyze the origin and development of the seed coat of the mycoheterotrophic orchid *Pogoniopsis schenckii* and to compare this development with that of other photosynthetic species of the family. Flowers and fruits at different stages of development were collected, and the usual methodology for performing anatomical studies, scanning microscopy, and transmission microscopy following established protocols. *P. schenckii* have ategmic ovules, while the other species are bitegmic. No evidence of integument formation at any stage of development was found through anatomical studies. The reduction of integuments found in the ovules could facilitate fertilization in this species. The seeds of *P. schenckii*, *Vanilla planifolia*, and *V. palmarum* have hard seed coats, while the other species have seed coats formed by the testa alone, making them thin and transparent. *P. schenckii,* in contrast to the other species analyzed, has a seed coat that originates from the nucellar epidermis, while in other species, the seed coat originates from the outer integument.

## Introduction

Flowers are highly variable structures, resulting in a great morphological diversity and a variety of adaptive processes in angiosperms (Endress, 1994; Friis et al., 2011). Variations in flower size and number of whorls, besides the presence or absence of fused floral parts, are caused by differences that occur during the development of floral organs. Plants exhibit open organization, which means that their organs are generally exposed, and that they do not have any organ or parts of organs internalized, with the exception of carpels (Endress, 2015). In turn, carpels can be free or united, becoming curved during their initial development, with edges getting closed or sealed when they are fully developed (Endress, 2006). While most floral organs are exposed, mainly due to the action of pollinating agents (e.g., animals, wind, and water), ovules are completely enclosed in the carpel a condition known as angiospermy (Endress, 2006).

Ovules are female reproductive structures that develop in the seeds (Bouman, 1984; Endress, 2011). Despite their relatively stable basic structure, ovules have a wide diversity of form, varying in terms of their position in the ovary, size, curvature, number and thickness of integument, funiculus length, and degree of vascularization (Endress, 2011). For angiosperms, there are records of bitegmic, unitegmic, and ategmic species (Bouman, 1984; Endress, 2011). Although most angiosperms are bitegmic, variation in the number and thickness of integuments can be observed at different taxonomic levels, such as in families and genera. For example, in Olacaceae, there are described bitegmic, unitegmic, and ategmic species (Brown et al., 2010). In Melastomataceae, ovules are bitegmic; however, in species of the same genus, the number of the outer integument layers can vary from two to many (Caetano et al., 2018).

Previous studies have described the main function of integuments as the delimitation of the micropyle, and protection to the embryo sac and embryo (Herrero, 2001); however, they may also have other functions in species of different families. For example, the inner epidermis of the inner integument can function as a secretory tissue, playing a role in the nutrition of the embryonic sac. This layer of cells is known as integumentary tapetum (Kapil and Tiwari, 1978). Another hypothesis is that the number of integument layers could be related to the fruit type and seed dispersal mode. A study performed with several species of Melastomataceae tried to confirm if there was a relationship between ovules with multiseriate outer integument and fleshy fruits (Caetano et al., 2018). The data obtained did not confirm this relation; however, ancestral state reconstruction shows a tendency for ovules with multiseriate outer integument to occur in fleshy fruit clades. Recent studies conducted with *Arabidopsis* show that the number of ovule integument layers is related to gene and hormone expression (Bencivenga et al., 2012; Gomez et al., 2016; Coen and Magnani, 2018) and may be responsible for the seed coat diversity observed in angiosperms. After fertilization, the integument layers go through different pathways to establish a protective barrier for the embryo (Windsor et al., 2000). There is an immense diversity in seed structure, such as size, color, texture, and shape; this diversity is related to dispersal and germination strategies (Boesewinkel and Bouman, 1984), and may have been initially determined by the arrangement and number of ovule integuments.

In most angiosperms, the formation of ovules is complete when anthesis starts. In Orchidaceae, however, a different pattern is observed, where in the development of ovules and their respective placental proliferation are conditioned to the pollination event (Swamy, 1949a). In general, orchids have low reproductive success because of low pollination rates (Cozzolino and Widmer, 2005). Thus, ovules will be produced only if there is guaranteed seed formation, in order to prevent unnecessary energy expenditure (Arditti, 1992). The formation of the integuments in orchids occurs simultaneously with the formation of the embryonic sac. To date, in most species studied, the embryonic sac is bitegmic (Swamy, 1949a; Yeung and Law, 1997; Mayer et al., 2011). However, four unitegmic species, which are all mycoheterotrophic, have been described in previous studies (Abe, 1976; Arekal and Karanth, 1981; Krawczyk et al., 2016; Li et al., 2016).

Mycoheterotrophic plants are aclorophyllated and are completely dependent on carbon available through their association with fungi throughout their life cycle (Leake, 1994). Recent phylogeny using plastid and mitochondrial genomes in Orchidaceae show that mycoheterotrophic species evolved several times independently (Li et al., 2019). In the family, 235 species with this condition are described (Merckx et al., 2013), and little is known about the reproductive process of these species. Owing to the importance of the seed coat in the life cycle of plants, and because it is considered a stable characteristic, understanding its structure and development can reveal information relevant to its functional aspects, such as dispersal and seed germination (Bouman, 1984; Windsor et al., 2000; Endress, 2011). Thus, the objective of this work was to analyze the origin and development of the seed coat of the mycoheterotrophic orchid *Pogoniopsis schenckii* Cogn, and to compare this development with that of other species in the family that have chlorophyll and present different mechanisms of seed dispersal, *Polystachya estrellensis* Rchb.f., *Elleanthus brasiliensis* Rchb.f., *Isochilus linearis* (Jacq) Barb.Rodr., and *Cleistes libonii* (Rchb. f.) Schltr.—species that exhibit anemochory, and *Vanilla planifolia* Jacks. ex Andrews and *Vanilla palmarum* (Salzm ex. Lindl.) Lindl.—species showing evidence of zoochory (Cribb, 1999). *Pogoniopsis schenckii* is an endemic mycoheterotrophic species found in the Brazilian Atlantic Forest. Prior studies indicate a tendency of reduction in the number of integuments in species of mycoheterotrophic plants, including orchids (Abe, 1976; Arekal and Karanth, 1981; Maas and Ruyters, 1986; Bouman et al., 2002; Endress, 2011; Krawczyk et al., 2016; Li et al., 2016). Thus, our hypothesis is that *P. schenckii* also exhibits reduction in the number of integuments, leading to a greater exposure of the ovule and simplification of the seed coat involving the embryo, which may facilitate the penetration of fungal hyphae. In this context, structural information on the reproductive organs of mycoheterotrophic species, especially *P. schenckii*, can contribute to the elucidation of processes related to the symbiosis between fungi and mycoheterotrophic species. In addition, since the mode of seed dispersal of *P. schenckii* is not known, characterization of the stages of development of its seeds can contribute to the understanding of the ecological interactions involved in the dispersal and colonization of new habitats.

## Material and Methods

### Species Studied and Literature Review

*Pogoniopsis schenckii* Cogn. -Epidendroideae- is aclorophyllated and remains underground for almost its entire life cycle. During its reproductive phase, a floral stem appears above ground level; afterwards, flowers and fruits develop. *Polystachya estrellensis* Rchb. f., *Elleanthus brasiliensis* Rchb. f., *Isochilus linearis* (Jacq) Barb. Rodr., belonging to the subfamily Epidendroideae, and *Cleistes libonii* (Rchb. f.) Schltr., *Vanilla planifolia* Jacks. ex Andrews, and *Vanilla palmarum* (Salzm ex. Lindl.) Lindl., belonging to the subfamily Vanilloideae, are photosynthetic species that are found in all Brazilian regions, and in different phytogeographical domains, such as the Cerrado, Atlantic Forest, and Amazon. Voucher specimens were deposited at the Herbarium of the University of Campinas (HUEC), Campinas, São Paulo, Brazil, and the registration numbers are: 196921, 205027, 161354, 197343, 205047, 205028, and 20502.

A literature review was carried out to verify the number of species that developed bitegmic ovules, and the number of species that developed unitegmic ovules. The following keywords were used to search publication databases: embryo development in Orchidaceae, embryology in Orchidaceae, and integuments in Orchidaceae.

#### Anatomic Analyses

To analyze the integument development freshly opened flowers off all species were collected. For *E. brasilliensis* and *V. palmarum* fruits from natural pollinations at different developmental stages were collected. For *P. schenckii* freshly opened flowers were marked and monitored. For the other species we carried out experimental self-pollination in flowers during the first day of anthesis. The flowers were then monitored and fruits at different stages of development were collected with 15, 20, 25, 30, 60, and 90 opening flower/days after pollination. All material was fixed in Karnovsky (Karnovsky, 1965), dehydrated in serial dilutions of ethanol, and were infiltrated with hydroxyethylmethacrylate (Gerrits and Smid, 1983). The samples were sectioned at 4 µm thickness using a Leica RM2245 rotary microtome, stained with Toluidine Blue 0.05% in phosphate buffer, pH 4.5 (Sakai, 1973), and mounted using Entellan^®^ synthetic resin (Merck^®^). The slides were analyzed under an Olympus BX51 optical microscope and photographed with an Olympus DP71 digital camera.

#### Scanning Electron Microscopy

Botanical material was fixed in Karnovsky’s solution (Karnovsky, 1965), dehydrated using a serial dilution of ethanol and critical point dried under carbon dioxide (CO_2_) in a Balzers model CPD 030 Critical Point Dryer. The material was then mounted on metal supports and coated with colloidal gold for 220 s on the Bal-Tec model SCD 050 equipment. Analysis and electron micrograph recordings were performed using a LEO VP 435 scanning electron microscope at 20 kV, at the Institute of Biology/UNICAMP.

#### Transmission Electron Microscopy

To analyze the changes occurring during the development of *P. schenckii* seed coat, ovules, and seeds at different stages of development were fixed using 2.5% glutaraldehyde in 0.2 M sodium cacodylate buffer, pH 7.25, for 24 h (Mc Dowel and Trump, 1976). Post-fixation was performed with 1.0% osmium tetroxide (OsO4) in sodium cacodylate buffer for 12 h in the dark (Gabriel, 1982). The material was dehydrated in a series of increasing concentrations of acetone solution and soaked in LR White^®^ resin according to the manufacturer’s instructions. The ultrafine sections were prepared with Leica ultramicrotome using diamond knife. The ultrafine sections were contrasted with uranyl acetate (Bozzola and Russel, 1998) and lead citrate (Hanaich et al., 1986), examined under a Philips EM 100 transmission electron microscope at 80 kV, and documented with Eastman Kodak 5302 (35 mm) film.

## Results

Literature review showed that 97 species of orchids of different subfamilies, 2 species of Vanilloideae, 8 species of Cypripedioideae, 31 species of Orchidoideae, and 56 species of Epidendroideae, had been evaluated till date. Regarding these species, 93 presented bitegmic ovules, and 4 presented unitegmic ovules (**Table 1** and **Supplementary Table 1**). With the 6 new species included in this study, a total of 103 species have been evaluated in terms of the type of ovule integuments.

**Table 1:** List of the type of integuments in species of Orchidaceae.

Species	Subfamily	Integument	Reference
*Cleistes libonii* (Rchb. f.) Schltr.	Vanilloideae	Biteg	Present study
*Vanilla palmarum* (Salzm ex. Lindl.) Lindl.	Vanilloideae	Biteg	Present study
*Vanilla planifolia* Jacks. ex Andrews	Vanilloideae	Biteg	Nishimura and Yukawa, 2010
*Vanilla imperialis* Kraenzl.	Vanilloideae	Biteg	Kodahl et al., 2015
*Paphiopedilum delenatii* Guillaumin	Cypripedioideae	Biteg	Lee and Yeung, 2012
*Cypripedium cordigerum* D. Don	Cypripedioideae	Biteg	Sood and Mohana Rao, 1988
*Cypripedium spectabile* (C. hirsutum Mill.)	Cypripedioideae	Biteg	Swamy, 1945
*Cypripedium parviflorum* Salisb.	Cypripedioideae	Biteg	Pace, 1907
*Cypripedium pubenscens* (Willd.)	Cypripediodeae	Biteg	Pace, 1907
*Cypripedium formosanum* Haiata	Cypripediodeae	Biteg	Lee et al., 2005
*Cypripedium macranthos* Sw.	Cypripediodeae	Biteg	Zeng et al., 2014
*Cypripedium japonicum* Thunb.	Cypripediodeae	Biteg	Liu et al., 2012
*Amitostigma kinoshitae* (Makino) Schltr.	Orchidoideae	Biteg	Abe, 1977
*Zeuxine gracilis* (Breda) Blume	Orchidoideae	Biteg	Gurudeva, 2011
*Zeuxine sulcata* Lindl.	Orchidoideae	Biteg	Swamy, 1946a
*Orchis aristata* Fisher	Orchidoideae	Biteg	Abe, 1972
*Platanthera tipuloides* Lindl. var. nipponica (Makino) Ohwi	Orchidoideae	Biteg	Abe, 1972
*Platanthera chlorantha* Custer (Rchb.)	Orchidoideae	Biteg	Abe, 1972
*Platanthera sachalinensis* Fr. Schm.	Orchidoideae	Biteg	Abe, 1972
*Peristylus spiralis* A. Rich	Orchidoideae	Biteg	Swamy, 1949a
*Peristylus stocksii* Krzl.	Orchidoideae	Biteg	Swamy, 1949a
*Dactylohiza maculata* (L.) Vermln.	Orchidoideae	Biteg	Fredrikson et al., 1988
*Herminium monorchis* (L.) R. Br.	Orchidoideae	Biteg	Fredrikson, 1990
*Spiranthes australis* Lindl.	Orchidoideae	Biteg	Maheshwari and Naraynaswami, 1951
*Spiranthes sinensis* (Pers.) Ames	Orchidoideae	Biteg	Lu-Han et al., 2016
*Habenaria platyphylla* Spr.	Orchidoideae	Biteg	Swamy, 1946b
*Habenaria rariflora* A. Rich.	Orchidoideae	Biteg	Swamy, 1946b
*Habenaria longicalcarata* A. Rich.	Orchidoideae	Biteg	Swamy, 1946b
*Habenaria decipiens* Wight.	Orchidoideae	Biteg	Swamy, 1946b
*Habenaria plantagenea* Lindl.	Orchidoideae	Biteg	Swamy, 1946b
*Habenaria longicornu* Lindl.	Orchidoideae	Biteg	Swamy, 1946b
*Habenaria marginata* Coleb.	Orchidoideae	Biteg	Swamy, 1946b
*Habenaria heyeneana* Lindl.	Orchidoideae	Biteg	Swamy, 1946b
*Habenaria viridiflora* R. Br.	Orchidoideae	Biteg	Swamy, 1946b
*Habenaria densa* Wall.	Orchidoideae	Biteg	Mohana Rao and Sood, 1979
*Habenaria galeandra* Hook. f.	Orchidoideae	Biteg	Sood, 1986
*Habenaria elisabethae* Duthie	Orchidoideae	Biteg	Sood, 1986
*Habenaria edgeworthii* Hook. f. ex. Collett	Orchidoideae	Biteg	Sood, 1986
*Habenaria radiata* (Thunb.) Spreng.	Orchidoideae	Biteg	Abe, 1972
*Habenaria sagittifera* (Reichb.) f.	Orchidoideae	Biteg	Abe, 1972
*Goodyera repens* (L.) R. Br.	Orchidoideae	Biteg	Sood, 1988
*Myrmechis japonica* (Reichb. f.) Br.	Orchidoideae	Biteg	Abe, 1972
*Gymnadenia camtschatica* Miyabe et Kudo	Orchidoideae	Biteg	Abe, 1972
*Pogoniopsis schenckii* Cogn.	Epidendroideae	Ateg	Present study
*Polystachya estrelensis* Rchb.f.	Epidendroideae	Biteg	Present study
*Isochilus linearis* (Jacq) Barb. Rodr.	Epidendroideae	Biteg	Present study
*Elleanthus brasiliensis* Rchb. f.	Epidendroideae	Biteg	Present study
*Coelogyne breviscapa* Lindl.	Epidendroideae	Biteg	Swamy, 1949a
*Coelogyne odorotissima* Lindl.	Epidendroideae	Biteg	Swamy, 1949a
*Calypso bulbosa* L.	Epidendroideae	Biteg	Law and Yeung, 1989
*Spathoglotis plicata* Bl.	Epidendroideae	Biteg	Swamy, 1949a
*Geodorum densiflorum* Schlechter.	Epidendroideae	Biteg	Swamy, 1949a
*Oncidium flexuosum* Sims	Epidendroideae	Biteg	Mayer et al., 2011
*Cymbidium sinense* (Andr.) Willd.	Epidendroideae	Biteg	Yeung, 1996
*Eulophia nuda* Lindl	Epidendroideae	Biteg	Swamy, 1949a
*Geodorum densiflorum* Schlechter.	Epidendroideae	Biteg	Swamy, 1949a
*Bulbophyllum mysorense* J. J. Smith.	Epidendroideae	Biteg	Swamy, 1949a
*Bulbophyllum neilgherrense* Wt. Ic. t.	Epidendroideae	Biteg	Swamy, 1949a
*Dendrobium barbatulum* Lindl.	Epidendroideae	Biteg	Swamy, 1949a
*Dendobrium haemoglossum* Thw.	Epidendroideae	Biteg	Swamy, 1949a
*Dendobrium microbulbon* A. Rich.	Epidendroideae	Biteg	Swamy, 1949a
*Dendobrium graminifolium* Wt. Ic. t.	Epidendroideae	Biteg	Swamy, 1949a
*Epidendrum variegatum* Hook	Epidendroideae	Biteg	Sharp, 1912
*Epidendrum ibaguense* Lindl.	Epidendroideae	Biteg	Yeung and Law, 1989
*Gastrodia elata* Blume	Epidendroideae	Uniteg	Abe, 1976; Li et al., 2016
*Gastrodia nantoensis*	Epidendroideae	Uniteg	Li et al., 2016
*Microstylis cylindrostachya* Reichb. F	Epidendroideae	Biteg	Sood, 1985
*Microstylis wallichii* Lindl.	Epidendroideae	Biteg	Sood and Mohana Rao, 1986
*Malaxis saprophyta* (King & Panting) Tang & F.T. Wang	Epidendroideae	Biteg	Sood, 1992
*Oberonia iridiflora* var. *denticulata* Hook	Epidendroideae	Biteg	Swamy, 1949a
*Epipactis atrorubens* (Hoffm.) Besser	Epidendroideae	Biteg	Fredrikson, 1992
*Epipactis helleborine* (L.) Crantz	Epidendroideae	Biteg	Fredrikson, 1992
*Epipactis palustris* (L.) Crantz	Epidendroideae	Biteg	Fredrikson, 1992
*Epipogium aphyllum* Sw.	Epidendroideae	Uniteg	Krawczyk et al., 2016
*Epipogium roseum* (D. Don) Lindl.	Epidendroideae	Uniteg	Arekal and Karanth, 1981
*Rhynchostylis retusa* Blume	Epidendroideae	Biteg	Swamy, 1949a
*Diplocentrum recurvum* Lindl.	Epidendroideae	Biteg	Swamy, 1949a
*Diplocentrum conjestrum* Wt. Ic. t.	Epidendroideae	Biteg	Swamy, 1949a
*Luisia teretrifolia* Gaud	Epidendroideae	Biteg	Swamy, 1949a
*Luisia teunifolia* Bl.	Epidendroideae	Biteg	Swamy, 1949a
*Cottonia peduncularis* Wt. Ic. t.	Epidendroideae	Biteg	Swamy, 1949a
*Saccolabium filiforme* Lindl.	Epidendroideae	Biteg	Swamy, 1949a
*Saccolabium jerdonianum* Reichb.	Epidendroideae	Biteg	Swamy, 1949a
*Saccolabium gracile* Lindl.	Epidendroideae	Biteg	Swamy, 1949a
*Saccolabium pulchellum* Fisher.	Epidendroideae	Biteg	Swamy, 1949a
*Saccolabium matsuran* Makino	Epidendroideae	Biteg	Abe, 1972
*Vanda spathulata* Spreng	Epidendroideae	Biteg	Swamy, 1949a
*Aerides cylindricum* Lindl.	Epidendroideae	Biteg	Swamy, 1949a
*Aerides ringens* Fisher.	Epidendroideae	Biteg	Swamy, 1949a
*Phalaenopsis* sp.	Epidendroideae	Biteg	Zhang and O’Neill, 1993
*Phalaenopsis amabilis* var. *formosa* Shimadzu	Epidendroideae	Biteg	Lee et al., 2008
*Eleorchis japonica* (A. Gray) F Maekawa	Epidendroideae	Biteg	Abe, 1972
*Bletia shepherdii* Hook.	Epidendroideae	Biteg	Sharp, 1912
*Phaius grandifolius* Lour.	Epidendroideae	Biteg	Sharp, 1912
*Phaius minor* Blume	Epidendroideae	Biteg	Abe, 1972
*Phaius tankervilliae* (Aiton ) Bl.	Epidendroideae	Biteg	Dong-mei et al., 2006
*Calanthe anistrifera* Reichb. f.	Epidendroideae	Biteg	Abe, 1972
*Calanthe discolor* Lindl.	Epidendroideae	Biteg	Abe, 1972
*Calanthe torifera* Schltr.	Epidendroideae	Biteg	Abe, 1972
*Ephippianthus schmidtii*	Epidendroideae	Biteg	Abe, 1972
*Liparis paradoxa* Reichb.	Epidendroideae	Biteg	Sood, 1989
*Liparis rostrata* Reichb. f.	Epidendroideae	Biteg	Sood, 1989
*Acianthera johannensis* (Barb Rodr) Pridgeon & M.W. Chase	Epidendroideae	Biteg	Duarte et al., 2019

Ateg, ategmic ovules; biteg, bitegmic ovules; uniteg, unitegmic ovules.

**Figure 1:**
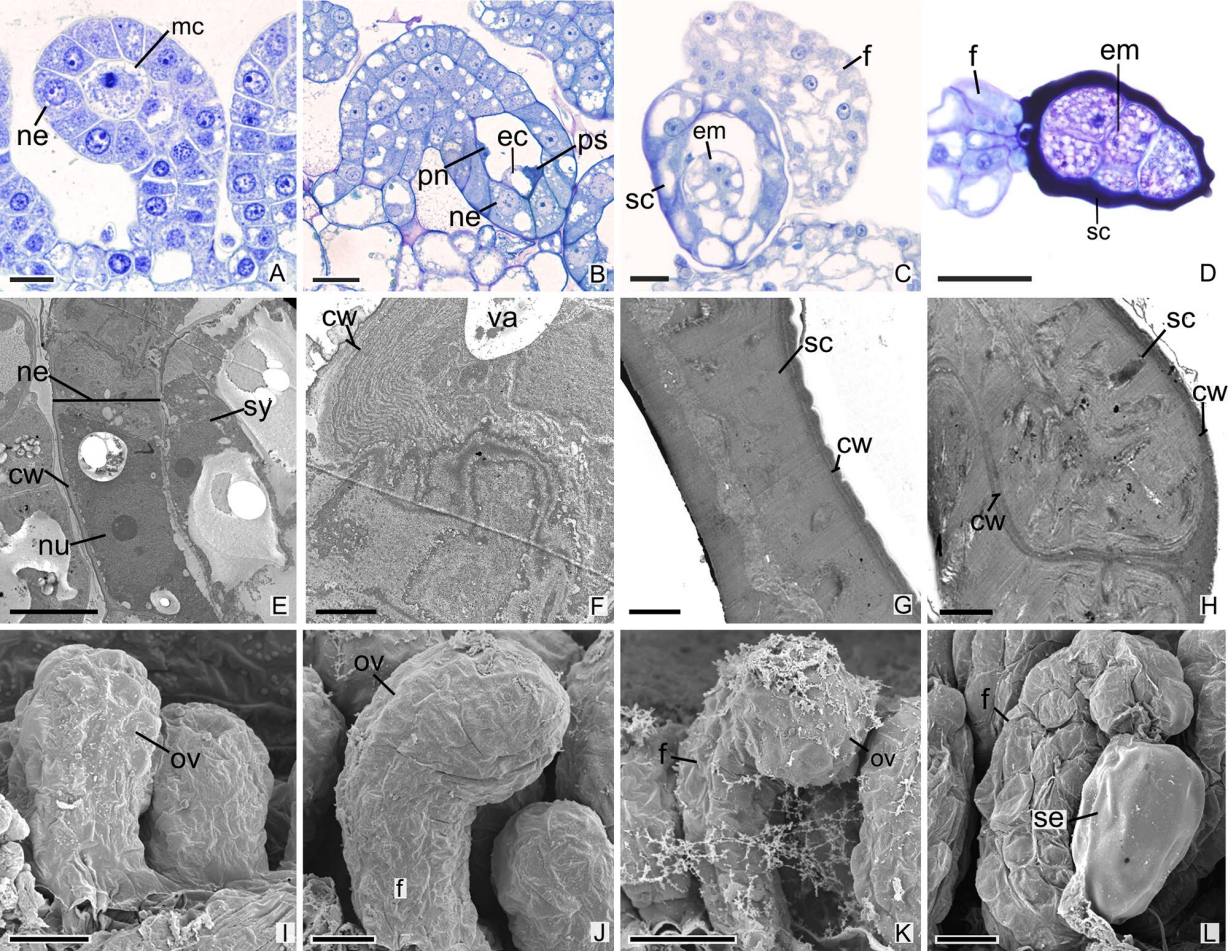
Longitudinal sections of ovules and seeds of *Pogoniopsis schenckii*. **(A)** Megaspore mother cell. Note that there is no formation of integuments. **(B)** Penetrated synergid, egg cell, and polar nuclei. **(C)** Young seed. **(D)** Embryo with five cells. **(E**–**H)** Transmission microscopy electromyography. **(E)** Embryo sac and nucellar epidermis. **(F)** Details of the nucellar epidermis which presents cells with thin walls and evident nucleus. **(G)** Seed coat that originates from the nucellar epidermis. **(H)** Details of the hard seed coat. Note their accumulation of substances that confers the cytoplasm a dense aspect. **(I**–**L)** Scanning microscopy electromyography. **(I**–**K)** Ovule in development. Note that there is no formation of integuments. **(L)** Seed. cw, cell wall; ec, egg cell; em, embryo; f, funiculus; mc, megaspore mother cell; ov, ovule; ne, nucellar epidermis; nu, nuclei; pn, polar nuclei; ps, penetrated synergid; sc, seed coat; se, seed; sy, synergid; va, vacuolo. Scale bars A, C, I = 20 µm; B, D, K, L = 50 µm; E, J = 10 µm; F–H = 2 µm.

All species analyzed in this study are teninucellate and bitegmic, except for *P. schenckii* (**Figures 1A–J**), which has an ategmic ovule. *P. schenckii* is the first species within Orchidaceae possessing this characteristic. There is no growth of integuments at any stage of development (**Figures 1A, B, I, J**). The embryonic sac is only covered by the nucellar epidermis, which exhibit cells with high metabolism, thin walls, and evident nucleus (**Figures 1A, B, E, F**). Owing to the absence of integuments, there is no micropyle formation in the ovule of the species (**Figures 1B, I, J, K**).

**Figure 2:**
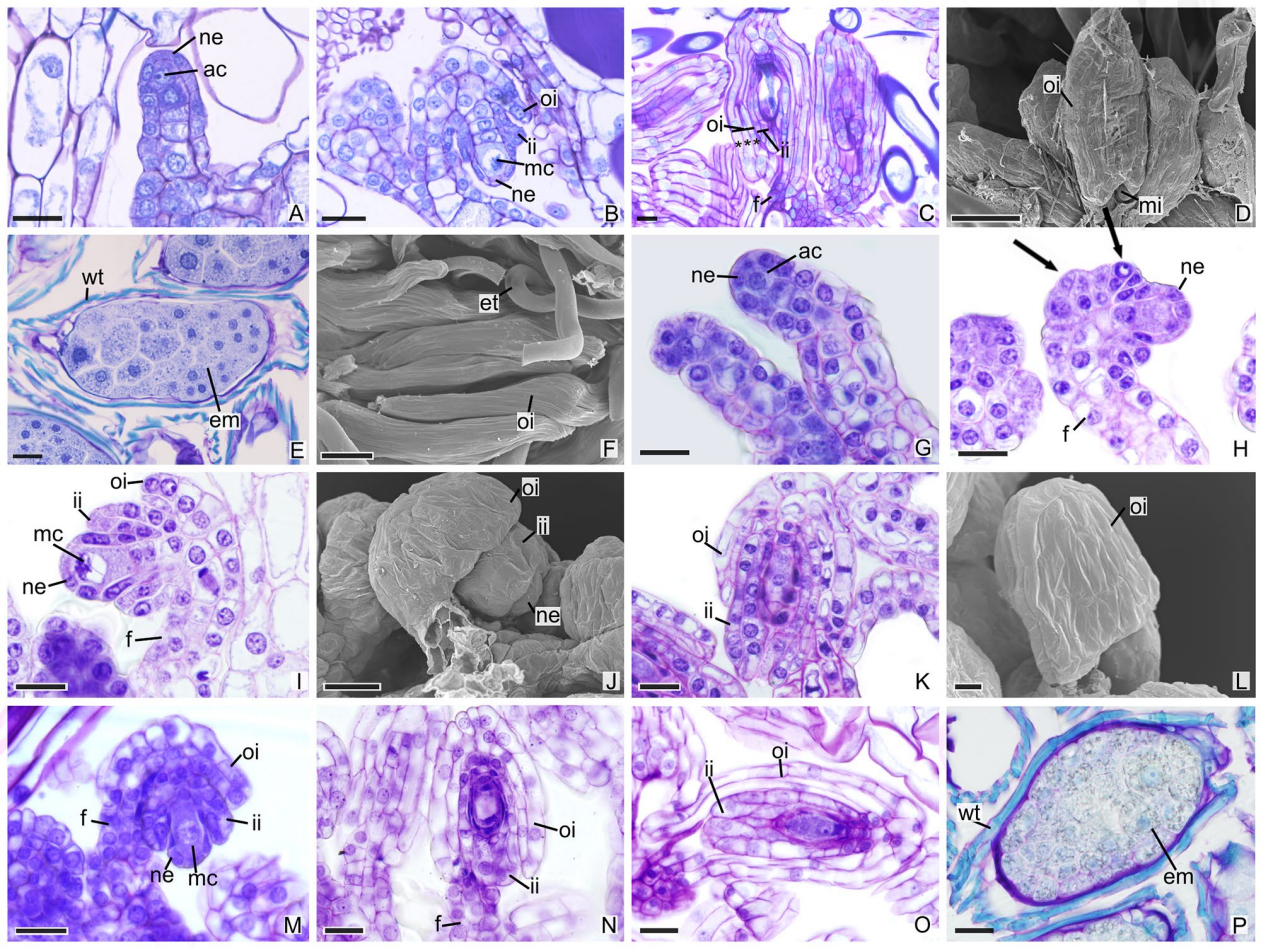
Longitudinal sections of ovules and seeds of Epidendroideae species. **(A**–**F)** Ovules and seeds of *Polystachya estrellensis*. **(D**, **F)** Scanning microscopy electromyography. **(A)** Differentiation of the initial archesporial cell. **(B)** Megaspore mother cell and formation of integuments. **(C)** Embryo sac. *indicates the outer integument with three layers. **(D)** Embryo sac. **(E**, **F)** Seed. **(G**–**L)** Ovules of *Elleanthus brasiliensis*. **(J**, **L)** Scanning microscopy electromyography. **(G)** Differentiation of the initial archesporial cell. **(H)** Formation of integuments indicated by arrows. **(I)** Megaspore mother cell. **(J**–**K)** Formation of integuments. **(L)** Embryo sac with the outer integument developed. **(M**–**P)** Ovules and seeds of *Isochilus linearis*. **(M)** Megaspore mother cell and formation of integuments. **(N**, **O)** Embryo sac with the integuments developed. P. Seed. ac, initial archesporial cell; em, embryo; f, funiculus; ii, inner integument; mc, megaspore mother cell; mi, micropyle; ne, nucellar epidermis; oi, outer integument; wt, wall thickening. Scale bars A–C; E; G-K; M-P = 20 µm; D, F = 50 µm; L = 10 µm.

**Figure 3:**
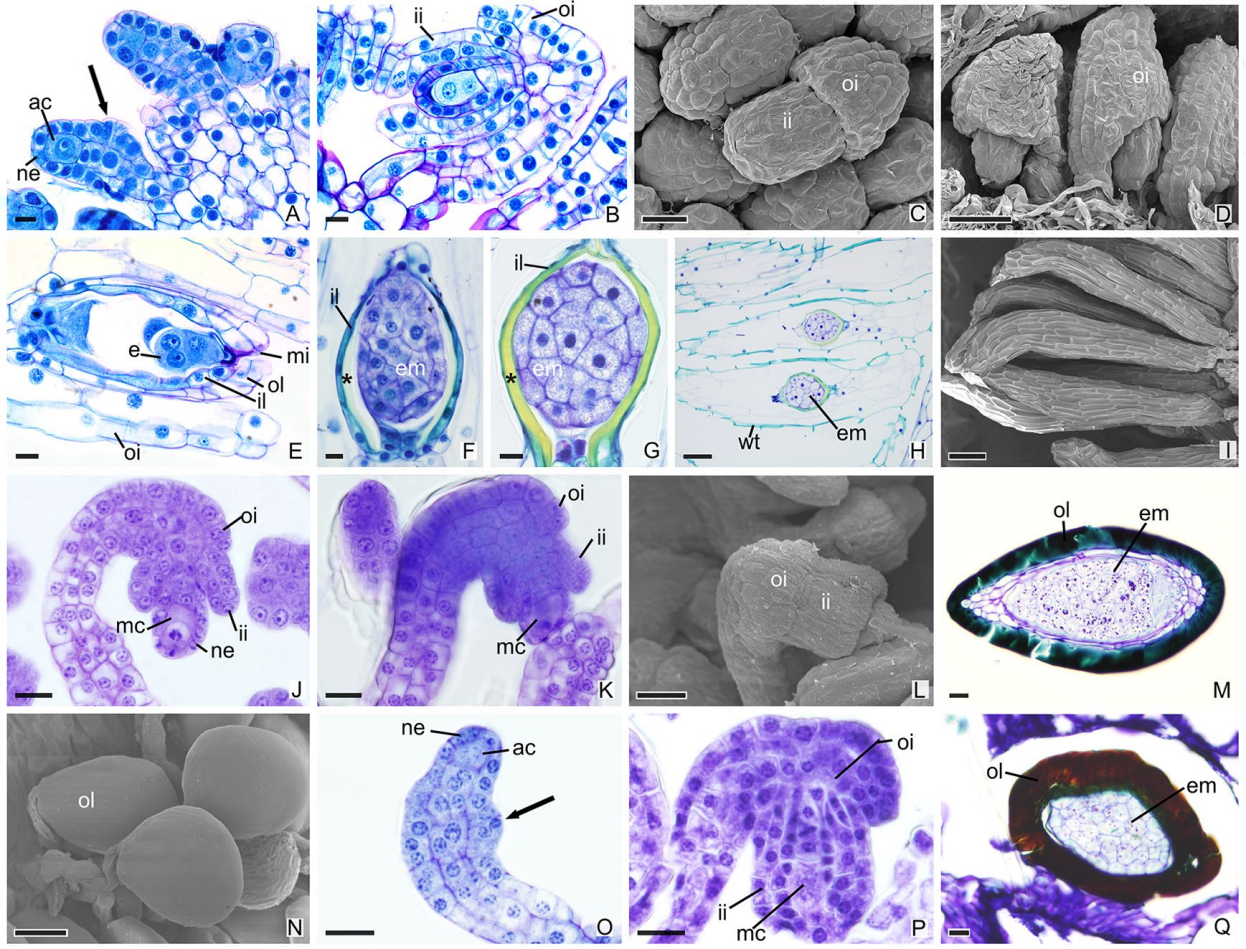
Longitudinal sections of ovules and seeds of Vanilloideae species. **(A**–**I)** Ovules and seeds of *Cleistes libonii*. **(C**, **D**, **I)** Scanning microscopy electromyography. **(A)** Differentiation of the initial archesporial cell. Arrows indicate the initial formation of integuments. **(B)** Megaspore mother cell and formation of integuments. **(C**, **D)** Development of integuments. **(E)** Initial development of embryo. **(F**–**G)** Embryo. *indicate the extracellular exudate. **(H**–**I)** Seeds. **(J**–**N)** Ovules and seeds of *Vanilla planifolia*. **(L**, **N)** Scanning microscopy electromyography. **(J**–**K)** Megaspore mother cell and formation of integuments. **(L)** Development of integuments. **(M**, **N)** Seeds with hard dark-colored coat. **(O**–**Q)** Ovules and seed of *Vanilla palmarum*. **(O)** Differentiation of the initial archesporial cell. Arrows indicate the initial formation of integuments.(P) Megaspore mother cell and formation of integuments. Note the outer integument with three layers. **(Q)** Seed with hard dark-colored coat. ac, initial archesporial cell; em, embryo; f, funiculus; ii, inner integument; il, inner layer of the inner integument; mc, megaspore mother cell; mi, micropyle; ne, nucellar epidermis; oi, outer integuments; ol, out layer of the inner integument; wt, wall thickening. Scale bars A, B, E–G, J–M, O–Q = 20 µm; C = 50 µm; D, H, N = 100 µm; I = 200 µm.

In other species, the development of integuments occurs from periclinal divisions in the epidermal cells located at the base of the ovules, which is seen in both the subfamily Epidendroideae (**Figures 2A–P**) and in the subfamily Vanilloideae (**Figures 3A–Q**). During ovule development, the inner integument grows and recovers the nucellar epidermis. In *P. estrellensis*, *E. brasiliensis*, *I. linearis,* and *C. libonii*, the outer integument grows and covers the inner integument (**Figures 2A–D, G–L, M–O** and **3A–E**). In *P. estrellensis*, the outer integument has three layers (**Figure 2C**). The initial development of the integuments in *V. planifolia* and *V. palmarum* show that the outer integument also has three layers (**Figure 3P**). In other species, the outer integument has two layers, and in all the species analyzed, the inner integument has two layers (**Figures 2M** and **3B**).

**Figure 4:**
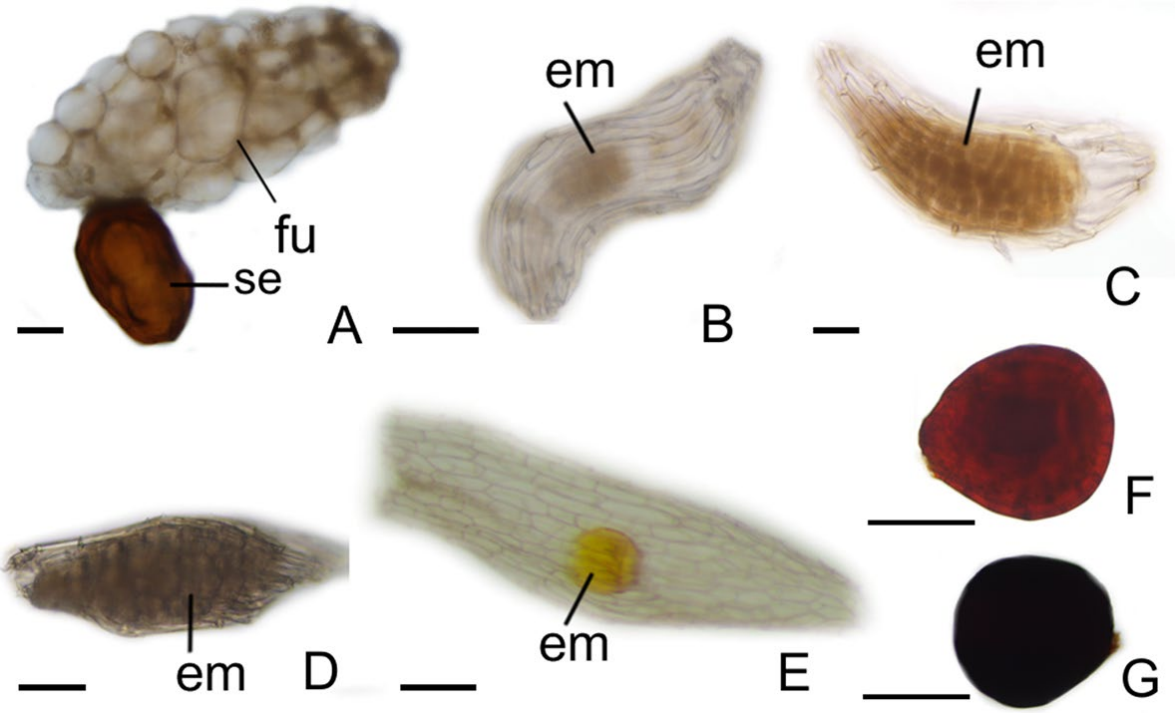
Aspects of seeds. **(A)** Pogoniopsis schenckii. **(B)** Polystachya estrellensis. **(C)** Elleanthus brasiliensis. **(D)** Isochilus linearis. **(E)** Cleistes libonii. **(F)** Vanilla planifolia. **(G)** Vanilla palmarum. em, embryo; fu, funiculus; se, seed . Scale bars A, B, C, D, E = 150 µm; F, G = 75 µm.

After fertilization, changes are observed in the integument of all species. In *P. schenckii* (i.e., about 25 days after the floral opening), the seed coat becomes hard and forms from the nucellar epidermis itself (**Figures 1C, D, G, H, L**). It is possible to observe that during development of the seed coat there is an accumulation of substances that confers the cytoplasm a dense aspect (**Figures 1G, H**). When mature, the seed presents a brown-colored integument (**Figure 4A**). In *P. estrellensis*, *E. brasiliensis,* and *I. linearis*, the inner integument is fully absorbed, and the outer integument undergoes elongation. In the outer integument, the inner layer is absorbed, and the outer layer gives rise to the seed testa, which, when mature, is impregnated with lignin and surrounds the embryo (**Figures 2D–F, P** and **4B–D**). In *C. libonii*, at the beginning of seed development (i.e., about 40 days after fertilization), the inner layer of the inner integument begins to possess a dense cytoplasm and an evident nucleus (**Figures 3E, F**). Sixty days after fertilization, it is possible to observe the presence of a natural yellow-colored secretion surrounding the embryo (**Figures 3G, H** and **4E**). In the mature seed, only the outer tegument and this secreted layer that covers the embryo remain as coat, whereas the inner layer of the internal integument is reabsorbed (**Figures 2E, P** and **4E**). This substance is probably secreted by the cells of the inner layer of the inner integument. In *V. planifoli*a and *V. palmarum*, the mature seed has a hard dark-colored coat (**Figures 3M, Q** and **4F, G**).

## Discussion

For the first time the presence of ategmic ovules, as observed in *P. schenckii*, are described in Orchidaceae. Bitegmic ovules are commonly in orchids (Swamy, 1949a), but a reduction in ovule integuments are commonly observed in mycoheterotrophic species, which are unitegmic (Tohda, 1967; Abe, 1976; Arekal and Karanth, 1981; Krawczyk et al., 2016; Li et al., 2016). Anatomical analyses show that the ovules of *P. schenckii* develop normally, and that there is no evidence of development of integument in the ovules of the species at all time points. In this way, the nucellar epidermis is responsible for surrounding the embryo sac, and in the mature seed, for surrounding the embryo. This result differs from the pattern found in other species analyzed in this study, which had seed coats originating from the outer integument.

Reduction of integuments occurs independently in different groups. They have been described in mycoheterotrophic species of Gentianales (Gentianaceae), parasite species of Santalales (Balanophoraceae, Loranthaceae, Olacaeae, and Santalaceae), and in a photosynthetic species of Aquifoliales (Cardiopteridaceae) (Maas and Ruyters, 1986; Bouman et al., 2002; Brown et al., 2010; Polli et al., 2016; Sato and Maria Gonzalez, 2016; Suaza-Gaviria et al., 2016; Tobe, 2016; Gonzalez et al., 2019). Molecular studies show that in ategmic ovules of Santalales, the genes associated with the expression of the integument are expressed in the periphery of the ovary, and that the reduction found in these species is the result of the fusion between the integument and the nucellus (Brown et al., 2010). In *P. schenckii*, the reduction of integuments leads to a total loss of the micropyle. However, this structural reduction does not seem to compromise reproduction, since the synergids continue to secrete substances for pollen tube attraction. The absence of integuments could facilitate the penetration of the synergids (**Figure 1B**) and subsequent fertilization. Mycoheterotrophic orchids have ovules with simpler structures, and the absence of a distinct micropyle is common in unitegmic species (Tohda, 1967; Abe, 1976; Arekal and Karanth, 1981; Li et al., 2016). The micropyle is responsible for directing the pollen tube; moreover, both the micropyle and secretions released by the synergid that promotes pollen tube attraction facilitate fertilization (Cheung and Wu, 2001; Okuda et al., 2009; Chen and Fang, 2016).

The differentiation of ovules in the species studied occurred after the stimulation of pollination, and the development of the integuments in *P. estrellensis*, *I. linearis*, *E. brasiliensis*, *C. libonii, V. planifolia,* and *V. palmarum* occurs simultaneously with the events of the megasporogenesis, as observed in other species of the family (Swamy, 1949a; Sood, 1985; Sood, 1986; Sood and Rao, 1986; Mayer et al., 2011; Li et al., 2016; Duarte et al., 2019). In most orchids, the outer integument has two layers of cells (Swamy 1949a; Wirth and Withner, 1959). However, in *P. estrellensis,* the outer integument was observed to have three layers, and the *Vanilla* species presented ovules with outer integuments that had three to four layers of cells in *V. imperialli*s, and four to six layers of cells in *V. planifolia* (Swamy, 1947; Nishimura and Yukawa, 2010; Kodahl et al., 2015). It is believed that the outer multiseriate integument in *Vanilla* would be related to the larger size of the seed found in the species of the genus (Kodahl et al., 2015).

Of all species analyzed, *P. schenckii*, *V. planifolia,* and *V. palmarum* have seeds with hard coat. Preliminary results show that in *P. schenckii*, dispersal in the species is very restricted (personal data). It was found to not be related to anemochory; moreover, dispersal by animals was not observed. Based on this, it seems that the hard coat has other unknown functions. Population genetics studies have been conducted, seeking to understand how this restricted dispersal can affect the dynamics of the populations of the species (Alves, unpublished data). Besides the hard coat *P. schenckii* presents seed with a large funiculus, differing from the other analyzed species. Preliminary analyzes show that the funiculus assists in the penetration of fungal hyphae after dispersion (personal data). Seeds with hard coat have also been described for other mycoheterotrophic orchids. For *Cyrtosia japonica*, seeds with coats originating from the outer integument and inner integument (Yang and Lee, 2014) are registered. In *C. japonica*, the seed presents an outer integument with four layers, and the outermost layer later becomes sclerified (Yang and Lee, 2014). It is suggested that the observed lignification protects the embryo when it passes through the alimentary tract of its dispersers (Rodolphe et al., 2011; Yang and Lee, 2014). In *Yonia japonica*, the seed also presents a lignified coat; however, the fruits and seeds of the species are dispersed by insects (Suetsugu, 2018). Similar to *Cyrtosia*, it is believed that the lignified seed coat in *Y. japonica* is an adaptation that protects the seed during digestion (Suetsugu, 2018).

It is assumed that *V. planifolia* and *V. palmarum* undergo endozoochory dispersal (Cribb, 1999; Kodahl et al., 2015). In these species, as in others belonging to Vanilloideae, seeds with hard coats exist as a strategy for the dispersal of the genus (Kodahl et al., 2015); in addition, hard coats would protect seeds that can be dispersed over long distances. In other species, seeds were observed to have a thin and transparent coat, which is seemingly a common feature in Orchidaceae. Seeds from Orchidaceae have small sizes, and are called “dust seeds” (Swamy, 1949b; Arditti and Ghani, 2000). The rather small size observed in orchid seeds was traditionally thought to be an adaptation to long-distance wind dispersal events (Arditti and Ghani, 2000). However, recent molecular studies have shown discordant patterns that show the orchid seeds ability to reach long distances (Cozzolino et al., 2003; Trapnell and Hamrick, 2004). Many other species present dispersal patterns limited to a few meters (Chung et al., 2004; Chung et al., 2005; Ren et al., 2017).

Dark-colored hard seed coats, as observed in *P. schenckii*, *V. planifolia*, and *V. palmarum*, have already been described for *Apostasia* (Swamy, 1947). Occurrence of phytomelanin deposition in Asparagales seeds is described in the literature (Dahlgren et al., 1985). Phytomelanin is a dark and insoluble pigment that is found in different parts of plants and exhibit distinct transport load and structural stability (Nicolaus et al., 1964; Cordero and Casadevall, 2017). Phytomelanin’s main function is to confer protection to different conditions, such as environmental variations, harmful radiation, extreme temperatures, and chemical and mechanical stress (Roulin, 2014; Cordero and Casadevall, 2017). The dark-colored integument in the species studied may result from phytomelanin deposition. However, studies are still needed to clarify this issue.

The results obtained show novelties in the development of the seed coat in Orchidaceae. *P. schenckii* has an ategmic ovule and has a hard seed coat that originates from the nucellar epidermis. Mycoheterotrophic plants have numerous modifications in their morphology, reproductive biology, and physiology (Leake, 1994; Bidartondo, 2005), the most prominent among loss of photosynthetic function and severe ruptures in the plastid genome (Graham et al., 2016). The genomic losses observed may be related not only to photosynthetic processes, but also to the absence of genes that present other functions, such as genes related to reproductive functions. Anatomical analyses show that there is no evidence of integument development in the ovules of *P. schenckii.* Thus, the reduction of the integuments found in the species may be due to the absence of gene expression, or even the absence of genes linked to the development of the integument; however, molecular studies are necessary to elucidate this issue.

## Data Availability Statement

The datasets generated for this study are available on request to the corresponding author.

## Author Contributions

MA carried the anatomical analysis, analysis of scanning and transmission microscopy, and writing the manuscript. FP supervised the work and writing the manuscript. MN carried anatomical analyses in *Cleistes libonii* and JM was responsible for collecting the material, carried anatomical analyses, writing the manuscript, and supervised the work.

## Funding

This study was financed in part by the Coordenação de Aperfeiçoamento de Pessoal de Nível Superior - Brasil (CAPES) - Finance Code 001. Juliana Lischka Sampaio Mayer thank FAPESP (2015/26479-6), FAEPEX 0944/14, CNPq (447453/2014-9), and CNPQ (310184/2016-9) for funding support.

## Conflict of Interest

The authors declare that the research was conducted in the absence of any commercial or financial relationships that could be construed as a potential conflict of interest.

## Supplementary Material

Supplementary Table 1 List of the type of integuments in species of Orchidaceae. Ateg = ategmic ovules; biteg = bitegmic ovules; uniteg = unitegmic ovules.
