# Supplementary material for: First Record of Ategmic Ovules in Orchidaceae Offers New Insights Into Mycoheterotrophic Plants
Source: Front Plant Sci. 2019 Nov 29;10:1447. doi: 10.3389/fpls.2019.01447 (PMC6895064; doi:10.3389/fpls.2019.01447)
Supplement: Supplementary Table 1 — List of the type of integuments in species of Orchidaceae. Ateg = ategmic ovules; biteg = bitegmic ovules; uniteg = unitegmic ovules. [file Table_1.doc]

| **Species** | **Subfamily** | **Integument** | **Reference** |
| --- | --- | --- | --- |
|  |  |  |  |
| *Cleistes libonii* (Rchb. f.) Schltr. | Vanilloideae | Biteg | Present study |
| *Vanilla palmarum* (Salzm ex. Lindl.) Lindl. | Vanilloideae | Biteg | Present study |
| *Vanilla planifolia* Jacks. ex Andrews | Vanilloideae | Biteg | Nishimura and Yukawa, 2010 |
| *Vanilla imperialis* Kraenzl. | Vanilloideae | Biteg | Kodahl et al., 2015 |
| *Paphiopedilum delenatii* Guillaumin | Cypripedioideae | Biteg | Lee and Yeung, 2012 |
| *Cypripedium cordigerum* D. Don | Cypripedioideae | Biteg | Sood and Mohana Rao, 1988 |
| *Cypripedium spectabile* (C. hirsutum Mill.) | Cypripedioideae | Biteg | Swamy, 1945 |
| *Cypripedium parviflorum* Salisb. | Cypripedioideae | Biteg | Pace, 1907 |
| *Cypripedium pubenscens* (Willd.) | Cypripediodeae | Biteg | Pace, 1907 |
| *Cypripedium formosanum* Haiata | Cypripediodeae | Biteg | Lee et al*.*, 2005 |
| *Cypripedium macranthos* Sw. | Cypripediodeae | Biteg | Zeng et al., 2014 |
| *Cypripedium japonicum* Thunb. | Cypripediodeae | Biteg | Liu et al., 2012 |
| *Amitostigma kinoshitae* (Makino) Schltr. | Orchidoideae | Biteg | Abe, 1977 |
| *Zeuxine gracilis* (Breda) Blume | Orchidoideae | Biteg | Gurudeva, 2011 |
| *Zeuxine sulcata* Lindl*.* | Orchidoideae | Biteg | Swamy, 1946a |
| *Orchis aristata* Fisher | Orchidoideae | Biteg | Abe, 1972 |
| *Platanthera tipuloides* Lindl. var. nipponica (Makino) Ohwi | Orchidoideae | Biteg | Abe, 1972 |
| *Platanthera chlorantha* Custer (Rchb.) | Orchidoideae | Biteg | Abe, 1972 |
| *Platanthera sachalinensis* Fr. Schm. | Orchidoideae | Biteg | Abe, 1972 |
| *Peristylus spiralis* A. Rich | Orchidoideae | Biteg | Swamy, 1949a |
| *Peristylus stocksii* Krzl. | Orchidoideae | Biteg | Swamy, 1949a |
| *Dactylohiza maculata* (L.) Vermln. | Orchidoideae | Biteg | Fredrikson, 1988 |
| *Herminium monorchis* (L.) R. Br. | Orchidoideae | Biteg | Fredrikson, 1990 |
| *Spiranthes australis* Lindl*.* | Orchidoideae | Biteg | Maheshwari and Naraynaswami, 1951 |
| *Spiranthes sinensis* (Pers.) Ames | Orchidoideae | Biteg | Lu-Han et al., 2016 |
| *Habenaria platyphylla* Spr. | Orchidoideae | Biteg | Swamy, 1946b |
| *Habenaria rariflora* A. Rich. | Orchidoideae | Biteg | Swamy, 1946b |
| *Habenaria longicalcarata* A. Rich. | Orchidoideae | Biteg | Swamy, 1946b |
| *Habenaria decipiens* Wight. | Orchidoideae | Biteg | Swamy, 1946b |
| *Habenaria plantagenea* Lindl. | Orchidoideae | Biteg | Swamy, 1946b |
| *Habenaria longicornu* Lindl. | Orchidoideae | Biteg | Swamy, 1946b |
| *Habenaria marginata* Coleb. | Orchidoideae | Biteg | Swamy, 1946b |
| *Habenaria heyeneana* Lindl. | Orchidoideae | Biteg | Swamy, 1946b |
| *Habenaria viridiflora* R. Br. | Orchidoideae | Biteg | Swamy, 1946b |
| *Habenaria densa* Wall. | Orchidoideae | Biteg | Mohana Rao and Sood, 1979 |
| *Habenaria galeandra*  Hook. f. | Orchidoideae | Biteg | Sood, 1986 |
| *Habenaria elisabethae* Duthie | Orchidoideae | Biteg | Sood, 1986 |
| *Habenaria edgeworthii* Hook. f. ex. Collett | Orchidoideae | Biteg | Sood, 1986 |
| *Habenaria radiata* (Thunb.) Spreng. | Orchidoideae | Biteg | Abe, 1972 |
| *Habenaria sagittifera* (Reichb.) f. | Orchidoideae | Biteg | Abe, 1972 |
| *Goodyera repens* (L.) R. Br. | Orchidoideae | Biteg | Sood, 1988 |
| *Myrmechis japonica* (Reichb. f.) Br. | Orchidoideae | Biteg | Abe, 1972 |
| *Gymnadenia camtschatica* Miyabe et Kudo | Orchidoideae | Biteg | Abe, 1972 |
| *Pogoniopsis schenckii* Cogn. | Epidendroideae | Ateg | Present study |
| *Polystachya estrelensis* Rchb.f*.* | Epidendroideae | Biteg | Present study |
| *Isochilus linearis* (Jacq) Barb. Rodr. | Epidendroideae | Biteg | Present study |
| *Elleanthus brasiliensis* Rchb. f. | Epidendroideae | Biteg | Present study |
| *Coelogyne breviscapa* Lindl. | Epidendroideae | Biteg | Swamy, 1949a |
| *Coelogyne odorotissima* Lindl. | Epidendroideae | Biteg | Swamy, 1949a |
| *Calypso bulbosa* L. | Epidendroideae | Biteg | Law and Yeung, 1989 |
| *Spathoglotis plicata* Bl. | Epidendroideae | Biteg | Swamy, 1949a |
| *Geodorum densiflorum* Schlechter. | Epidendroideae | Biteg | Swamy, 1949a |
| *Oncidium flexuosum* Sims | Epidendroideae | Biteg | Mayer et al., 2011 |
| *Cymbidium sinense* (Andr.) Willd. | Epidendroideae | Biteg | Yeung et al., 1996 |
| *Eulophia nuda* Lindl | Epidendroideae | Biteg | Swamy, 1949a |
| *Geodorum densiflorum* Schlechter. | Epidendroideae | Biteg | Swamy, 1949a |
| *Bulbophyllum mysorense* J. J. Smith. | Epidendroideae | Biteg | Swamy, 1949a |
| *Bulbophyllum neilgherrense* Wt. Ic. t. | Epidendroideae | Biteg | Swamy, 1949a |
| *Dendrobium barbatulum* Lindl. | Epidendroideae | Biteg | Swamy, 1949a |
| *Dendobrium haemoglossum* Thw. | Epidendroideae | Biteg | Swamy, 1949a |
| *Dendobrium microbulbon* A. Rich. | Epidendroideae | Biteg | Swamy, 1949a |
| *Dendobrium graminifolium* Wt. Ic. t. | Epidendroideae | Biteg | Swamy, 1949a |
| *Epidendrum variegatum* Hook | Epidendroideae | Biteg | Sharp, 1912 |
| *Epidendrum ibaguense* Lindl. | Epidendroideae | Biteg | Yeung and Law, 1989 |
| *Gastrodia elata* Blume | Epidendroideae | Uniteg | Abe, 1976/Li et al., 2016 |
| *Gastrodia nantoensis* | Epidendroideae | Uniteg | Li et al., 2016 |
| *Microstylis cylindrostachya* Reichb. F | Epidendroideae | Biteg | Sood, 1985 |
| *Microstylis wallichii* Lindl. | Epidendroideae | Biteg | Sood and Mohana Rao, 1986 |
| *Malaxis saprophyta* (King & Panting) Tang & F.T. Wang | Epidendroideae | Biteg | Sood, 1992 |
| *Oberonia iridiflora* var. *denticulata* Hook | Epidendroideae | Biteg | Swamy, 1949a |
| *Epipactis atrorubens* (Hoffm.) Besser | Epidendroideae | Biteg | Fredrikson, 1992 |
| *Epipactis helleborine* (L.) Crantz | Epidendroideae | Biteg | Fredrikson, 1992 |
| *Epipactis palustris* (L.) Crantz | Epidendroideae | Biteg | Fredrikson, 1992 |
| *Epipogium aphyllum* Sw. | Epidendroideae | Uniteg | Krawczyk et al., 2016 |
| *Epipogium roseum* (D. Don) Lindl. | Epidendroideae | Uniteg | Arekal and Karanth, 1981 |
| *Rhynchostylis retusa* Blume | Epidendroideae | Biteg | Swamy, 1949a |
| *Diplocentrum recurvum* Lindl. | Epidendroideae | Biteg | Swamy, 1949a |
| *Diplocentrum conjestrum* Wt. Ic. t. | Epidendroideae | Biteg | Swamy, 1949a |
| *Luisia teretrifolia* Gaud | Epidendroideae | Biteg | Swamy, 1949a |
| *Luisia teunifolia* Bl*.* | Epidendroideae | Biteg | Swamy, 1949a |
| *Cottonia peduncularis* Wt. Ic. t. | Epidendroideae | Biteg | Swamy, 1949a |
| *Saccolabium filiforme* Lindl*.* | Epidendroideae | Biteg | Swamy, 1949a |
| *Saccolabium jerdonianum* Reichb. | Epidendroideae | Biteg | Swamy, 1949a |
| *Saccolabium gracile* Lindl. | Epidendroideae | Biteg | Swamy, 1949a |
| *Saccolabium pulchellum* Fisher. | Epidendroideae | Biteg | Swamy, 1949a |
| *Saccolabium matsuran* Makino | Epidendroideae | Biteg | Abe, 1972 |
| *Vanda spathulata* Spreng | Epidendroideae | Biteg | Swamy, 1949a |
| *Aerides cylindricum* Lindl. | Epidendroideae | Biteg | Swamy, 1949a |
| *Aerides ringens* Fisher. | Epidendroideae | Biteg | Swamy, 1949a |
| *Phalaenopsis* sp. | Epidendroideae | Biteg | Zhang and O'Neill, 1993 |
| *Phalaenopsis amabilis* var. *formosa* Shimadzu | Epidendroideae | Biteg | Lee et al., 2008 |
| *Eleorchis japonica* (A. Gray) F Maekawa | Epidendroideae | Biteg | Abe, 1972 |
| *Bletia shepherdii* Hook. | Epidendroideae | Biteg | Sharp, 1912 |
| *Phaius grandifolius* Lour. | Epidendroideae | Biteg | Sharp, 1912 |
| *Phaius minor* Blume | Epidendroideae | Biteg | Abe, 1972 |
| *Phaius tankervilliae* (Aiton ) Bl. | Epidendroideae | Biteg | Dong-mei et al., 2006 |
| *Calanthe anistrifera* Reichb. f. | Epidendroideae | Biteg | Abe, 1972 |
| *Calanthe discolor* Lindl*.* | Epidendroideae | Biteg | Abe, 1972 |
| *Calanthe torifera* Schltr. | Epidendroideae | Biteg | Abe, 1972 |
| *Ephippianthus schmidtii* | Epidendroideae | Biteg | Abe, 1972 |
| *Liparis paradoxa* Reichb. | Epidendroideae | Biteg | Sood, 1989 |
| *Liparis rostrata* Reichb. f. | Epidendroideae | Biteg | Sood, 1989 |
| *Acianthera johannensis* (Barb Rodr) Pridgeon & M.W. Chase | Epidendroideae | Biteg | Duarte et al., 2019 |
